# Dapagliflozin‐lowered blood glucose reduces respiratory Pseudomonas aeruginosa infection in diabetic mice

**DOI:** 10.1111/bph.13741

**Published:** 2017-03-09

**Authors:** Annika Åstrand, Cecilia Wingren, Audra Benjamin, John S Tregoning, James P Garnett, Helen Groves, Simren Gill, Maria Orogo‐Wenn, Anders J Lundqvist, Dafydd Walters, David M Smith, John D Taylor, Emma H Baker, Deborah L Baines

**Affiliations:** ^1^ Respiratory, Inflammation and Autoimmunity Innovative Medicines Research UnitAstraZeneca GothenburgMölndalSweden; ^2^ Institute for Infection and ImmunitySt George's, University of LondonLondonUK; ^3^ Mucosal Infection & Immunity Group, Section of VirologyImperial College London, St Mary's CampusLondonUK; ^4^ Cardiovascular & Metabolic Diseases Innovative Medicines Research UnitAstraZeneca GothenburgMölndalSweden

## Abstract

**Background and Purpose:**

Hyperglycaemia increases glucose concentrations in airway surface liquid and increases the risk of pulmonary *Pseudomonas aeruginosa* infection. We determined whether reduction of blood and airway glucose concentrations by the anti‐diabetic drug dapagliflozin could reduce P. aeruginosa growth/survival in the lungs of diabetic mice.

**Experimental Approach:**

The effect of dapagliflozin on blood and airway glucose concentration, the inflammatory response and infection were investigated in C57BL/6J (wild type, WT) or leptin receptor‐deficient (db/db) mice, treated orally with dapagliflozin prior to intranasal dosing with LPS or inoculation with P. aeruginosa. Pulmonary glucose transport and fluid absorption were investigated in Wistar rats using the perfused fluid‐filled lung technique.

**Key Results:**

Fasting blood, airway glucose and lactate concentrations were elevated in the db/db mouse lung. LPS challenge increased inflammatory cells in bronchoalveolar lavage fluid from WT and db/db mice with and without dapagliflozin treatment. P. aeruginosa colony‐forming units (CFU) were increased in db/db lungs. Pretreatment with dapagliflozin reduced blood and bronchoalveolar lavage glucose concentrations and P. aeruginosa CFU in db/db mice towards those seen in WT. Dapagliflozin had no adverse effects on the inflammatory response in the mouse or pulmonary glucose transport or fluid absorption in the rat lung.

**Conclusion and Implications:**

Pharmacological lowering of blood glucose with dapagliflozin effectively reduced P. aeruginosa infection in the lungs of diabetic mice and had no adverse pulmonary effects in the rat. Dapagliflozin has potential to reduce the use, or augment the effect, of antimicrobials in the prevention or treatment of pulmonary infection.

**Abbreviations**
ASL: airway surface liquid
BALF: bronchoalveolar lavage fluid
CFU: colony forming units
CF: cystic fibrosis
COPD: chronic obstructive pulmonary disease
db/db: leptin receptor deficient
SGLT: sodium coupled glucose transporter
WT: wild type

## Tables of Links

**Table:** 

**TARGETS**	
**Catalytic receptors** ^*a*^	**Transporters** ^*b*^
Leptin receptor	SGLT1
	SGLT2

**LIGANDS**
Dapagliflozin
LPS
Phlorizin

These Tables list key protein targets and ligands in this article which are hyperlinked to corresponding entries in http://www.guidetopharmacology.org, the common portal for data from the IUPHAR/BPS Guide to PHARMACOLOGY (Southan *et al*., 2016), and are permanently archived in the Concise Guide to PHARMACOLOGY 2015/16 (^*a,b*^Alexander *et al*., 2015a,b).

## Introduction

People with diabetes mellitus are at increased risk of, and have worse outcomes from, lower respiratory tract infections compared to those without diabetes mellitus. This is a particular problem in chronic lung disease, where diabetes is a common comorbidity. In people with chronic obstructive pulmonary disease (COPD), diabetes is associated with an increased likelihood and frequency of exacerbations (Kinney *et al.,* 2014), and increased duration of hospital stay and mortality from exacerbations (Gudmundsson *et al.,* 2006; Parappil *et al.,* 2010). In those with cystic fibrosis (CF), diabetes is an independent risk factor for pulmonary exacerbations (Jarad and Giles, 2008; Sawicki *et al.,* 2013) and for failure of intravenous or oral antibiotic treatment (Briggs *et al.,* 2012; Parkins *et al.,* 2012). In both COPD and CF, poor glycaemic control is positively associated with exacerbation frequency (Franzese *et al.,* 2008; Kupeli *et al.,* 2010).

An important mechanism whereby diabetes mellitus drives respiratory infection is through disruption of airway glucose homeostasis. In health, the glucose concentration of fluid lining human airways (airway surface liquid, ASL) is ~0.4 mM, 12.5 times lower than blood glucose concentrations (Baker *et al.,* 2007). Hyperglycaemia increases ASL glucose concentrations by threefold in healthy lungs and 10‐fold in chronic lung disease (Baker *et al.,* 2007). Increased ASL glucose concentrations predispose to respiratory infection, both by promoting the growth of pathogenic organisms that use glucose as a carbon source, particularly P. aeruginosa and S. aureus, and by suppressing host immunity. In both cell culture and animal lung models, elevation of blood glucose concentrations increases ASL glucose concentrations, which in turn drives respiratory infection (Garnett *et al.,* 2013b; Gill *et al.,* 2016). For example, P. aeruginosa (PAO1 strain) bacterial counts were higher in lung homogenates from leptin receptor deficient (db/db) and leptin‐deficient (ob/ob) diabetic mice, streptozotocin‐treated mice and alloxan‐treated diabetic rats than in non‐diabetic controls 6 h after respiratory inoculation (Pezzulo *et al.,* 2011; Gill *et al.,* 2016; Oliveira *et al.,* 2016). In humans, diabetes is associated with increased isolation of gram negative organisms in sputum from COPD patients and increased risk of lung colonization with P. aeruginosa in patients with CF (Loukides and Polyzogopoulos, 1996; Leclercq *et al.,* 2014).

Airway glucose homeostasis therefore represents a new treatment target in the prevention and treatment of respiratory infection that has the potential to reduce the use, or augment the effect, of antimicrobials. ASL glucose concentrations could be reduced by lowering blood glucose, reducing airway epithelial permeability to glucose or increasing glucose uptake by airway epithelial cells (Garnett *et al.,* 2012). Acute (48 h) metformin treatment reduced epithelial permeability to glucose by increasing expression of tight junction proteins (Patkee *et al.,* 2016) and decreased P. aeruginosa and S. aureus growth in the lungs of diabetic mice, despite being of insufficient duration to lower blood glucose (Garnett *et al.,* 2013a; Gill *et al.,* 2016). Sodium‐glucose co‐transporter isoform 2 (SGLT2) inhibitors are a relatively new class of anti‐diabetic drug that lower blood glucose by increasing renal excretion of glucose and, unlike metformin, do not appear to have off‐target effects in the lung (Madaan *et al.,* 2016). The primary aim of our study was to determine whether reduction of blood glucose by treatment with the SGLT2 inhibitor dapagliflozin could reduce ASL glucose and P. aeruginosa infection in the lungs of diabetic mice. Our secondary aims were to determine the effects of dapagliflozin on inflammation in the mouse lung, glucose transport and fluid absorption in the rat lung, so as to assess the pulmonary effects of this drug.

## Methods

### Animals

Male db/db mice, 14–15 weeks old (BKS.Cg‐m+/+Lepr^db^/J (db/db) C57BL/6J) (Charles River, Italy), average weight 49.7 ± 0.5 g and wild type (WT) C57BL/6J (24.0 ± 3.0 g) mice were used in the study. WT and db/db mice were allocated upon arrival into groups using restricted randomization so that average body weights were similar between the groups. Based on power calculations using data from similar studies 7–10 animals were used per group to detect meaningful differences. Treatment groups were blinded during result assessment and in some but not all data analyses. Results from studies repeated using the same procedures were pooled where possible. There was no significant loss of animals with any treatment. However, there were occasional unexplained losses of animals during the study which contributed to the difference in numbers (*n*) given per group. Male Wistar rats (Charles River Laboratories, Kent, UK), average weight 375.9 ± 19.4 g were randomly allocated to treatment groups of four animals based on previous data. Animals were housed in pathogen‐free facilities in cages with wooden chips, shredded paper, gnaw sticks and plastic houses, which were maintained at 21 ± 2°C with 55 ± 15% relative humidity and 12 h light/dark cycle. Water and food (RM3 pellet from Lantmännen, Sweden or RM1 expanded pellets from SDS, UK) were available *ad libitum.* Body weights in fed state were recorded during the course of the study to follow the wellbeing of the animals. Experiments were terminated if body weight decreased by 15% and/or if animals showed signs of distress, such as decreased movement, abnormal posture, dull eyes or piloerection.

### LPS challenge model

LPS challenge of 48 h versus no challenge (indicated as time 0) was carried out in C57BL/6J and db/db mice, with or with out dapagliflozin treatment (see below). LPS from P. aeruginosa (Sigma‐Aldrich, UK) was diluted in aqueous solution to give 0.0875 μg·g^−1^ mouse in 50 μL (based on the average weight of the group) and given by intranasal dosing at time 0. Animals were anaesthetised with isoflurane 4–5% (O_2_ 1.2 L·min^−1^) prior to administration of the LPS solution to one nostril, which was subsequently inhaled naturally. Mice were then returned to their cages when they had regained consciousness.

### Infection model

Db/db and WT C57BL/6 J mice were anaesthetised with isoflurane 4–5% (O_2_ 1.2 L·min^−1^) prior to intranasal infection with vehicle or 10^5^ colony forming units (CFU) of log phase P. aeruginosa (PAO1) in 100 μL. Mice were then returned to their cages when they had regained consciousness. Bronchoalveolar lavage fluid (BALF) was obtained from inoculated lungs 24 h later (see below). Lungs were then removed and homogenized by passage through 100 μm cell strainers. Bacterial CFU were determined in untreated BALF and lung homogenate by serial dilution on Luria broth agar (Sigma‐Aldrich, UK).

### Blood and BALF collections

Blood was collected from the vena saphena of conscious mice for glucose evaluation after 4 h of fasting. Animals were killed by an i.p. overdose of 0.2 mL pentobarbital (100 mg·mL^−1^). The lungs of each animal were subjected to bronchoalveolar lavage. In brief, the trachea was exposed and a catheter was inserted and secured with a silk suture. Three volumes of 0.3 mL saline were instilled, gently aspirated, pooled and weighed. There were occasions where BALF collection was impaired and sufficient samples volumes could not be obtained for analysis.

### BALF glucose, lactate and cell analysis

The BALF was centrifuged at 314x*g*, 10 min, 4°C. The supernatant was used to measure glucose and lactate concentration on the ABX Pentra 400 (Horiba ABX Medical, Kyoto, Japan) according to the manufacturer's protocol. The pellet was re‐suspended in 0.25 mL of PBS, and the total and differential cell count was performed using SYSMEX XT‐1800i Vet that uses fluorescent flow cytometry technology to differentiate between cell types (SYSMEX, Kobe, Japan). For the infection studies, BALF was treated with red blood cell lysis buffer before centrifugation at 200x*g* for 5 min. Cells were resuspended in RPMI medium with 10% FCS, and viable cell numbers were determined by trypan blue exclusion. For differential cell counts, 100 μL of cells from BALF and the lung homogenate were centrifuged onto glass slides, air dried and fixed in methanol before staining of with haematoxylin and eosin**.** Cell count is expressed as the number of cells mL^−1^ of recovered BALF. At termination, blood from behind the eye was collected in EDTA tubes and blood glucose was measured directly using Accu‐check (Roche, Bromma, Sweden). Plasma lactate was assayed using the ABX Pentra 400.

### Treatment with dapagliflozin

Treatment groups were given a daily oral dose of either vehicle (sterile water) or dapagliflozin (1 mg·kg^−1^) for 4 (LPS study) or 7 days (infection study) at a volume of 0.2 mL per mouse. Dapagliflozin/vehicle was administered just prior to the LPS challenge and 4 h before the P. aeruginosa infection.

Dapagliflozin concentrations in acetonitrile precipitated plasma samples were determined by LC–MS/MS. A gradient elution on a C18 column was used with acetonitrile/formic acid as the mobile phase system. The mass spectrometer operated in a positive/negative switching mode. Dapagliflozin plasma concentrations were 542 ± 83 nM, *n* = 10, which is comparable to maximum plasma concentrations recorded in people (100‐150 ng mL^−1^) (Yang *et al.,* 2013; Tirucherai *et al.,* 2016).

### Perfused fluid filled rat lung

Rats were terminally anaesthetised with i.p. injections of 75 mg·kg^−1^ ketamine (100 mg·mL^−1^)/1 mg·kg^−1^ medetomidine (1 mg·mL^−1^). Tracheotomy was performed, the rats ventilated with air (Harvard Rodent ventilator) and the chest opened in the midline. The animal was then treated with heparin (0.1 mL, 10 000 U mL^−1^), cannulated via the pulmonary artery and left ventricle, and the lungs perfused with a solution containing 3% BSA, 117 mM NaCl, 2.68 mM KCl, 1.25 mM MgSO_4_, 1.82 mM CaCl_2_, 20 mM NaHCO_3_, 5.55 mM glucose and 12 mM HEPES. The time of loss of circulation to the lung was ~10–20 s. The perfusate (100 mL) was maintained at 38°C, 95% O_2_/5% CO_2_ and circulated with a perfusion pressure of 7–8 mmHg and venous negative return pressure. Once perfusion was established, ventilation was stopped and the lung lumen filled with perfusate solution (15 mL·kg^−1^ body weight) with the exclusion of glucose. After a 40 min mixing period to degas the lung, the BALF was sampled (150 μL) every 10 min and the concentration of glucose was measured using an Analox GM9D glucose analyser (Analox Instruments Ltd). At 80 min, dapagliflozin (100 nM) or the sodium glucose co‐transporter isoform 1 and 2 (SGLT1/2) inhibitor phlorizin (100 μM) was added to the BALF, and further samples were taken at 10 min intervals up to 150 min to determine the specificity of dapagliflozin and/or any detrimental off‐target effects. Perfusion and venous pressures and perfusate flow rates as well as osmolality of the perfusate were monitored during the course of the experiment.

All experiments were performed under licence from the United Kingdom Home Office in accordance with the Animals (Scientific Procedures) Act 1986, amended 2012 or were approved by the local Ethical committee in Gothenburg (184‐2012). Animal studies are reported in compliance with the ARRIVE guidelines (Kilkenny *et al*., 2010; McGrath and Lilley, 2015).

### Statistical analysis

Values are reported as the mean ± SEM. Statistical analysis was performed using ANOVA tests followed by Bonferroni's multiple comparison *post hoc* tests (GraphPad Prism) or Student's *t*‐test (only if F achieved *P* < 0.05 and there was no significant variance in homogeneity). *P* values of <0.05 were considered statistically significant. The data and statistical analysis comply with the recommendations on experimental design and analysis in pharmacology (Curtis *et al.,* 2015).

## Results

### Diabetic db/db mice display elevated BALF glucose and lactate concentrations

**Figure 1:**
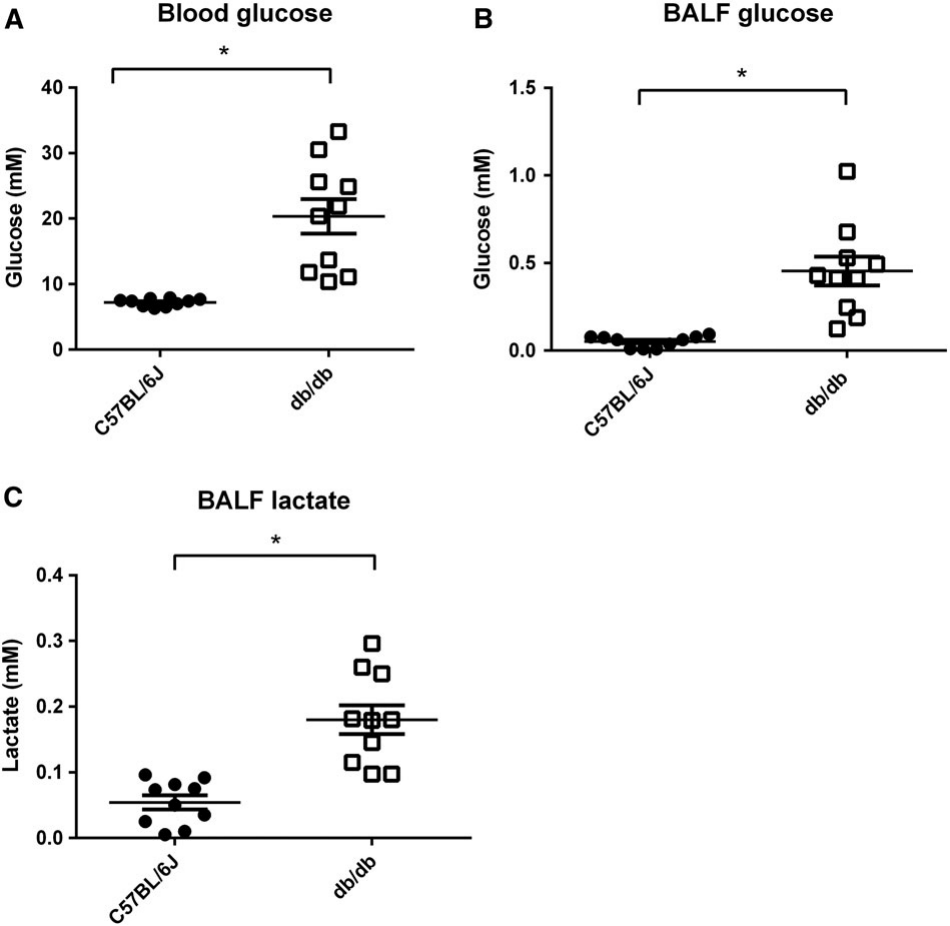
Airway glucose and lactate concentrations are elevated in hyperglycaemic mice. (A) Blood glucose, (B) BALF glucose and (C) BALF lactate all shown as mM and plotted for individual C57BL/6J WT or db/db mice (all *n* = 10). The horizontal lines indicate mean values ± SEM. Statistically different from WT or db/db treated with saline, * *P* < 0.05.

Fasting blood glucose concentrations were higher in db/db than C57BL/6J WT mice (20.36 ± 2.6, *n* = 10 compared to 7.20 ± 0.0.18 mM, *P* < 0.05, *n* = 10, Figure 1A) as were BALF glucose concentrations (0.45 ± 0.08 compared to 0.05 ± 0.01 mM, *P* < 0.05, *n* = 10 respectively, Figure 1B). Fasting lactate concentration was significantly higher in the BALF from db/db mice than from WT (0.18 ± 0.02 compared to 0.05 ± 0.01 mM, *P* < 0.05, *n* = 10 respectively, Figure 1C).

### Dapagliflozin reduces both fasting blood glucose and BALF glucose concentrations in db/db mice

**Figure 2:**
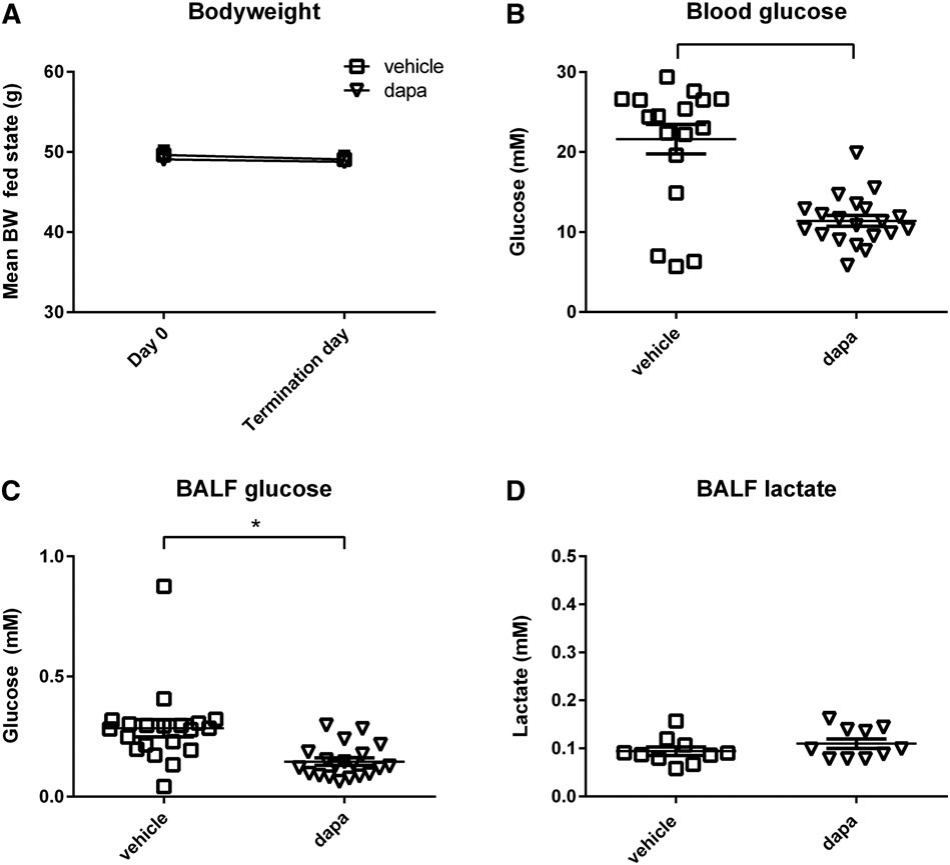
Treatment with dapagliflozin reduces both blood and airway glucose. (A) Body weight (BW; *n* = 10), (B) fasting blood glucose (*n* = 18 and *n* = 20), (C) BALF glucose (both *n* = 20) and (D) BALF lactate (*n* = 10). Body weight is shown as mean ± SEM at the start and end of the dapagliflozin treatment protocol. All others are plotted as individual db/db mice treated with vehicle or dapagliflozin (dapa). The horizontal lines indicate mean values ± SEM. Statistical difference between groups is indicated * *P* < 0.05.

**Figure 3:**
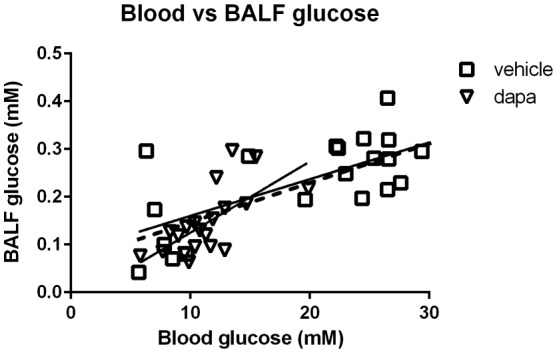
Dapagliflozin does not adversely affect the relationship between blood glucose and BALF glucose concentration. Values for blood glucose and BALF glucose concentration plotted for individual untreated (*n* = 20) or dapagliflozin pretreated db/db mice (*n* = 21). Lines of linear regression were significantly different from 0 (*r* ^2^ = 0.3, *P* < 0.05 and *r* ^2^ = 0.5, *P* < 0.05) respectively (solid lines), but not different from each other and a single line of regression (broken line) could be plotted for all data (*r* ^2^ = 0.5, *P* < 0.05, *n* = 41).

Treatment of db/db mice with dapagliflozin for 4 days had no significant effect on body weight (*n* = 10, Figure 2A). Dapagliflozin significantly reduced fasting blood glucose concentration in db/db mice (from 21.61 ± 1.86 to 11.40 ± 0.69 mM, *P* < 0.05, *n* = 18 and *n* = 20 respectively, Figure 2B). Dapagliflozin reduced the BALF glucose concentration of db/db mice (from 0.28 ± 0.04 to 0.15 ± 0.01 mM, *P* < 0.05, *n* = 20 respectively, Figure 2C). Dapagliflozin had no effect on the lactate concentration in the BALF of db/db mice (*n* = 10 respectively, Figure 2D). The positive relationship between blood glucose and BALF glucose in db/db mice was not altered by dapagliflozin. Lines of linear regression were significantly different from 0 (*r* ^2^ = 0.3, *P* < 0.05 and *r* ^2^ = 0.5, *P* < 0.05) respectively, but not different from each other (Figure 3). It was noted however, that data from one individual with a blood glucose of 19.90 mM and BALF glucose of 0.22 mM may have skewed the data. If this point was removed, the data analysis indicated that dapagliflozin promotes a lower BALF glucose concentration for any corresponding blood glucose than in untreated db/db mice with the slope of the line and x intercept closer to that of untreated WT mice (data not shown). This would infer that dapagliflozin had a beneficial effect on ASL glucose homeostasis additional to its action on blood glucose concentration. However, we could not find any experimental justification to remove this point. Dapagliflozin had no effect on body weight, blood glucose or BALF glucose concentration in WT mice (*n* = 10, data not shown).

### Lowering blood glucose with dapagliflozin reduces P. aeruginosa infection in db/db mouse lung

**Figure 4:**
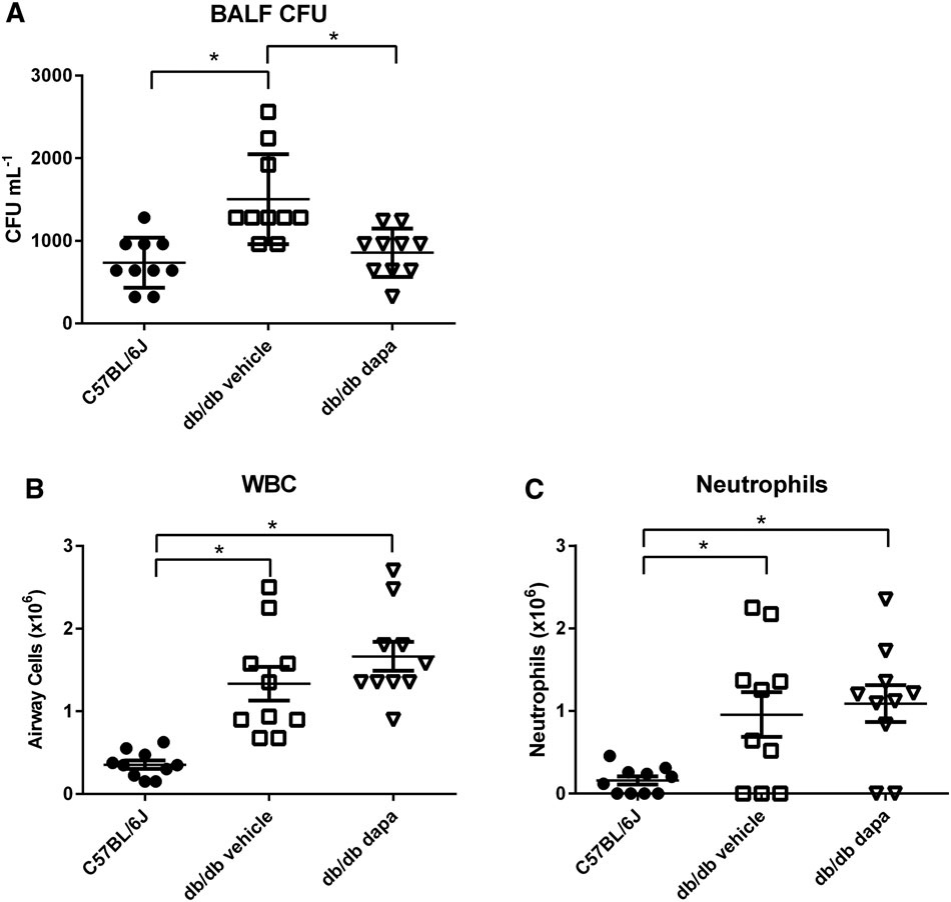
Dapagliflozin treatment reduces airway bacterial load. C57BL/6J WT or db/db mice pretreated for 7 days with vehicle or dapagliflozin were infected intranasally with P. aeruginosa. (A) P. aeruginosa CFU, (B) total WBC, (C) neutrophils in BALF. Data are plotted for individual animals 24 h after infection (all *n* = 10). The horizontal lines indicate mean values ± SEM. Statistical difference between groups is indicated *P < 0.05.

There was a significant increase in P. aeruginosa CFU recovered from the BALF of db/db mice compared to WT (1504 ± 172 and 736 ± 96 CFU mL^−1^, *P* < 0.05, *n* = 10 respectively, Figure 4A). Pretreatment with dapagliflozin reduced bacteria in BALF of db/db mice to that seen in WT (856 ± 92 CFU mL^−1^, *P* < 0.05, *n* = 10, Figure 4A). Dapagliflozin had no effect on CFU in WT (*n* = 10, data not shown) or survival in any group (*n* = 10, data not shown). Dapagliflozin did not alter the inflammatory response to P. aeruginosa infection. White blood cells (WBC) were significantly increased in db/db and dapagliflozin‐treated db/db mice (*P* < 0.05, *n* = 10 respectively) compared to WT, and the elevation was predominantly due to an increase in neutrophils (Figure 4B,C). Based on these observations, we suggest that dapagliflozin reduced bacterial load through its action on blood and airway glucose.

### Diabetic db/db mice exhibit increased airway inflammation that is not modified by dapagliflozin

**Figure 5:**
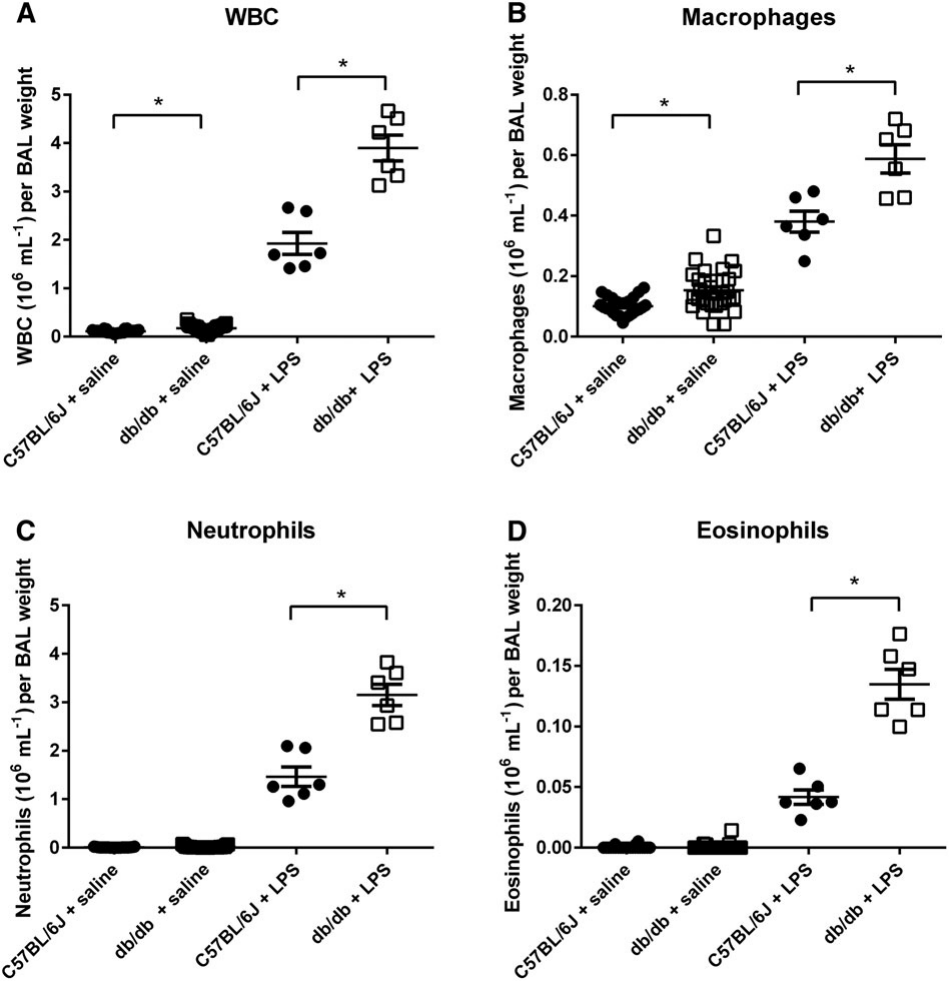
Hyperglycaemic animals have greater neutrophil response to LPS. (A) Total WBC, (B) macrophages, (C) neutrophils and (D) eosinophils all shown as ×10^6^ mL^−1^ and plotted for individual C57BL/6J WT or db/db mice treated (*n* = 24 and *n* = 32 respectively) with saline or LPS (both *n* = 6). The horizontal lines indicate mean values ± SEM. Statistical differences between groups are indicated * *P* < 0.05.

Reduction of inflammation is a treatment target in diabetes. We therefore explored whether dapagliflozin treatment could modify underlying inflammation in db/db mice and/or the acute response to an inflammatory stimulus (identified by increased inflammatory cells and lactate concentration in BALF) to ensure that it did not promote inflammation and to see if it could reduce inflammation. To do this, we used LPS from P. aeruginosa to mimic the bacterial insult. Without LPS treatment, db/db mice had higher numbers of WBC in the BALF than WT mice (0.17 ± 0.01 compared to 0.11 ± 0.01 × 10^6^ cells mL^−1^ weight, *P* < 0.05, *n* = 32 and 24 respectively, Figure 5A). This was predominantly due to increased macrophages (Figure 5B, *n* = 32).

Treatment of WT and db/db with intranasal LPS elicited an increase in inflammatory cells in BALF (all *n* = 6, Figure 5). The response to LPS in db/db mice was more robust than that of WT mice and was characterized by increased numbers of neutrophils, macrophages and eosinophils (Figure 5C,D). These data indicate that db/db mice had more baseline inflammatory cells in the lungs than WT mice and elicited a more robust neutrophil inflammatory response to LPS stimulation.

**Figure 6:**
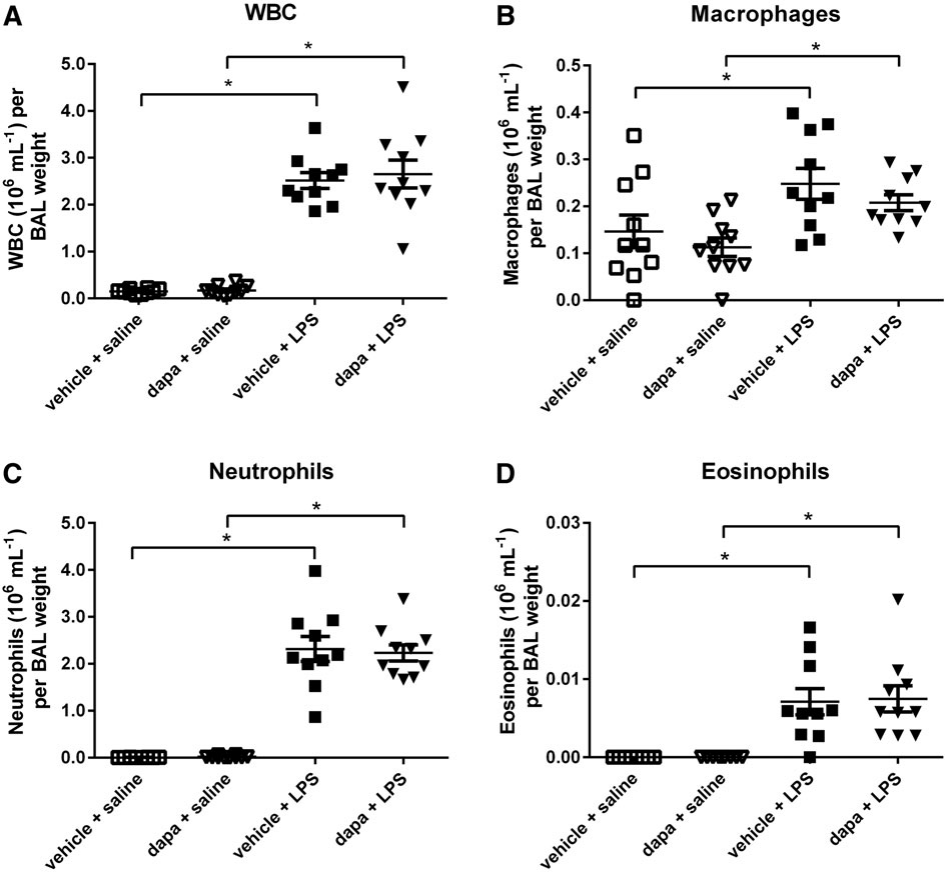
Dapagliflozin treatment does not reduce airway inflammatory cells. (A) Total WBC, (B) macrophages, (C) neutrophils and D) eosinophils all shown as ×10^6^ mL^−1^ and plotted for individual vehicle‐treated db/db mice or db/db mice pretreated with dapagliflozin (dapa) or LPS or dapa + LPS (all *n* = 10). The horizontal lines indicate mean values ± SEM. Statistical differences between groups are indicated * *P* < 0.05

Pretreatment with dapagliflozin had no effect on inflammatory cells in the BALF of db/db (*n* = 10, Figure 6A–D). Dapagliflozin also had no effect on the LPS‐induced increase in total WBC, neutrophils, macrophages or eosinophils numbers in the BALFs of db/db mice, *n* = 10 respectively (Figure 6A–D).

**Figure 7:**
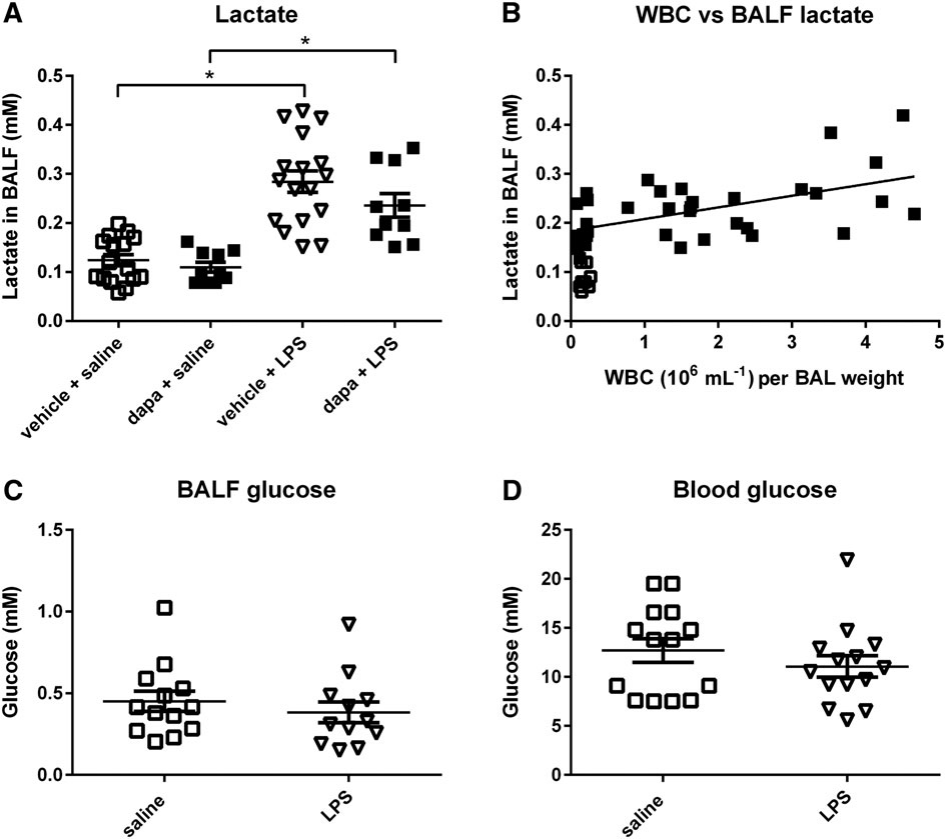
Dapagliflozin treatment does not change BALF lactate concentration. **(**A) Vehicle‐treated db/db mice (*n* = 16) or db/db mice pretreated with dapagliflozin (dapa) (*n* = 17) or LPS (*n* = 10) or dapa + LPS (*n* = 10). (B) Lactate concentration (mM) versus WBC plotted for individual vehicle‐treated (*n* = 10) or LPS‐treated (*n* = 40) db/db mice. BALF glucose (C) and blood glucose (D) concentrations are also shown. The horizontal lines indicate mean values ± SEM. Statistical differences between groups are indicated * *P* < 0.05.

BALF lactate concentration was increased in LPS‐treated db/db mice (0.12 ± 0.01, *n* = 16 to 0.28 ± 0.02 mM, *P* < 0.05, *n* = 17, Figure 7A). As expected, the number of WBC positively correlated with lactate concentration in the BALF of db/db mice (*r* ^2^ = 0.3, *P* < 0.05, (Figure 7B). Dapagliflozin had no effect on the LPS‐induced rise in lactate concentration in the BALF and concentrations remained elevated at 0.24 ± 0.02 mM, *n* = 10 (Figure 7C). Therefore, whilst dapagliflozin pretreatment lowered blood and airway glucose, it had no effect on the elevated inflammation seen in db/db animals. Treatment of db/db mice with LPS had no effect on blood (*n* = 14) or BALF glucose concentration (*n* = 12, Figure 7A,B).

### SGLT2 inhibitor dapagliflozin has no detrimental effect on lung glucose and fluid absorption

**Figure 8:**
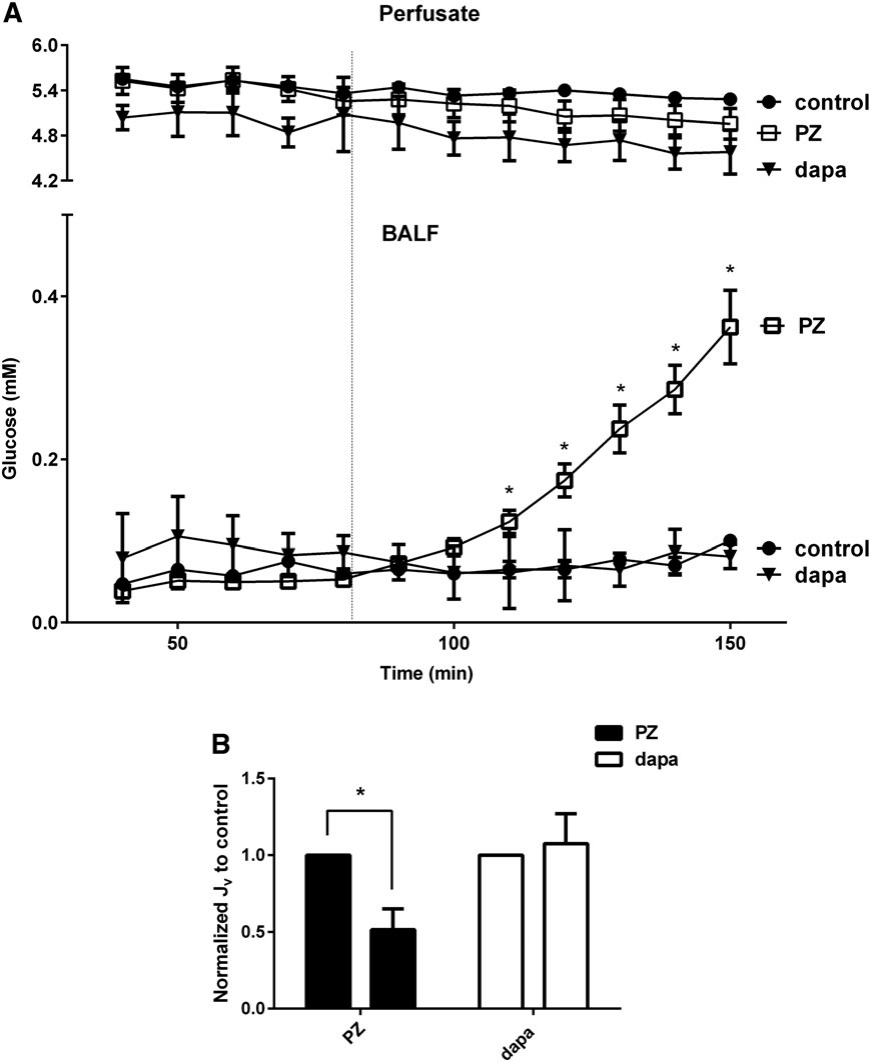
Dapagliflozin does not directly modify glucose transport or fluid homeostasis in the lung. (A) Upper axes, perfusate glucose concentration, Lower axes, BALF glucose concentration in the lungs of rats treated with vehicle (control), or phlorizin (PZ) or dapagliflozin (dapa) over an experimental time course of 150 min. A baseline was established over the first 40 min and vehicle or drugs were added as shown by the vertical dotted line. (B) Normalized Jv for vehicle (control) or phlorizin (PZ) or dapagliflozin (dapa) in the same experiment as described above. Data are shown as mean ± SEM. Significantly different from corresponding control samples, * *P* < 0.05.

To confirm the specificity of dapagliflozin as an inhibitor of SGLT2, without effect on the function of SGLT1, we measured BALF glucose in perfused, fluid‐filled rat lungs, with and without addition of dapagliflozin (SGLT2‐specific inhibitor) or phlorizin (SGLT1/2 inhibitor) to the lung instillate. There was no significant change in lung liquid glucose concentration in the presence of dapagliflozin (0.11 ± 0.02 mM at the end of control sampling to 0.15 ± 0.07 nM at the end of treatment sampling, *n* = 4) or lung liquid absorption rate (control rate: −0.02 ± 0.00 mL·min^−1^·g^−1^ dry lung weight to treatment rate: −0.02 ± 0.00 mL·min^−1^·g^−1^ dry lung weight, *n* = 4). Phlorizin significantly increased lung liquid glucose (0.05 ± 0.01 mM at the end of control sampling to 0.36 ± 0.05 mM at the end of treatment sampling; *P* < 0.05, *n* = 6, Figure 8A) and significantly decreased lung liquid absorption rate (control rate: −0.02 ± 0.00 mL·min^−1^·g^−1^ dry lung weight to treatment rate: −0.01 ± 0.00 mL·min^−1^·g^−1^ dry lung weight; *P* < 0.05, *n* = 6, Figure 8B). These data demonstrate that SGLT1, but not SGLT2, mediates Na^+^/glucose transport in the rat lung and that dapagliflozin has no effect on this process.

## Discussion

We found that reducing blood glucose by treatment with the SGLT2 inhibitor dapagliflozin reduced both BALF glucose concentrations and P. aeruginosa load in the lungs of leptin receptor‐deficient db/db diabetic mice. To our knowledge, this is the first study to demonstrate that control of blood glucose using an oral hypoglycaemic agent can prevent pulmonary infection, potentially by limiting movement of glucose into airway secretions.

We propose that dapagliflozin exerts its anti‐infective effect by restricting glucose availability for P. aeruginosa growth/survival in the lung through a reduction in blood and ASL glucose concentrations. In support of this, genetic impairment of sugar transport pathways in P. aeruginosa and S. aureus that limited sugar uptake had a similar effect on bacterial growth/survival in the lung to that of dapagliflozin (Pezzulo *et al.,* 2011; Garnett *et al.,* 2014; Gill *et al.,* 2016). Reduction of airway glucose in the distal mouse lung by manipulation of glucose transport pathways also reduced bacterial load (Oliveira *et al.,* 2016). Furthermore, we previously showed that treatment with the biguanide metformin reduced lung epithelial permeability and glucose flux into the lung lumen without affecting blood glucose concentration and inhibited the growth/survival of S. aureus and P. aeruginosa in diabetic mice (Garnett *et al.,* 2013a; Patkee *et al.,* 2016). Our new data indicate that lowering blood glucose concentration and consequently the gradient for glucose diffusion into the lung lumen has a similar effect to reducing epithelial permeability and glucose flux, resulting in restriction of bacterial growth/survival in the lungs. As dapagliflozin and metformin have different modes of action, it could be speculated that combined therapy would further reduce ASL glucose and infection. We have no evidence that insulin or insulin‐sensitizing effects of metformin and other type II diabetic drugs would further modify ASL glucose, although other beneficial effects cannot be ruled out.

These findings have potentially important clinical relevance and implications for human health. Diabetes mellitus affects ~25% people with COPD (Wells and Baker, 2013) and ~50% adults with CF (Li *et al.,* 2016), in whom it is associated with increased exacerbation rate (Kinney *et al.,* 2014) and increased sputum colonization with gram negatives*/* P. aeruginosa (Loukides and Polyzogopoulos, 1996; Leclercq *et al.,* 2014). Several very small studies in patients with chronic lung disease indicate that treatment with oral hypoglycaemics and/or insulin can reduce exacerbation rate and alter sputum microbiology (Lanng *et al.,* 1994; Rinne *et al.,* 2015). SGLT2 inhibitors have established application in the treatment of type 2 diabetes in COPD (NICE, 2013) and potential application as an insulin adjunct in the treatment of the insulin‐deficient diabetes seen in CF (Argento and Nakamura, 2016). Further investigation of the effect of dapagliflozin on chronic P. aeruginosa infection and the safety and efficacy of treatment with this drug in co‐morbid diabetes and respiratory disease are now required. This together with progress to a clinical investigation will determine whether blood glucose control with dapagliflozin can reduce P. aeruginosa load/colonization and/or exacerbation rate in patients with chronic lung disease.

This study has also generated important lung safety data. Dapagliflozin had no effect on inflammatory cell numbers and associated lactate concentration in the airways of mice prior to, or after, an acute pro‐inflammatory challenge. This indicates that dapagliflozin did not impair the inflammatory response required to clear infection. Inflammatory cells and lactate were increased in the lungs of untreated db/db mice and exposure to pro‐inflammatory stimuli promoted a characteristically neutrophilic response that was more robust compared to WT animals (Lu *et al.,* 2006; Vernooy *et al.,* 2010; Yano *et al.,* 2012). This difference in inflammatory response was not reported for hyperglycaemic GK^+/−^ or streptozotocin‐treated mice (Hunt *et al.,* 2014; Gill *et al.,* 2016) and indicates that lack of leptin receptor signalling and/or insulin‐resistance are more likely than glucose to mediate this effect in db/db mice (Lu *et al.,* 2006; Park *et al.,* 2009; Vernooy *et al.,* 2010; Yano *et al.,* 2012). Increased inflammatory status is a hallmark of diabetic patients and reduction of systemic inflammation has been proposed as a treatment target (Maiorino *et al.,* 2017). Although, there is no data from human studies yet (Scheen *et al.,* 2015), inhibition of SGLT1/2 was reported to improve systemic neutrophil phagocytosis in db/db mice (Yano *et al.,* 2012) and treatment with the SGLT2 inhibitor empagliflozin for 8 weeks (compared to 4 days used in this study), improved insulin sensitivity (Kern *et al.,* 2016). Thus, whether a more prolonged treatment with dapagliflozin would aid resolution of inflammation or improve inflammatory cell function requires further study.

In the well‐characterized perfused, fluid‐filled rat lung model, we found that dapagliflozin had no adverse effects on fluid absorption or lung glucose concentrations. The SGLT1 isoform is expressed in alveolar epithelium, where it contributes to sodium, glucose and fluid absorption (Bodega *et al.,* 2010). Phlorizin, an inhibitor of SGLT1/2, reduced lung liquid and glucose absorption, increasing luminal glucose concentrations with potential adverse effects of pulmonary oedema and increased bacterial proliferation (Oliveira *et al.,* 2016). Dapagliflozin, which has ~200‐fold selectivity for SGLT2 over SGLT1 (Han *et al.,* 2008) had no effect on lung fluid or glucose absorption, providing further evidence for pulmonary safety of this drug and no function of SGLT2 in the lung.

We conclude that dapagliflozin reduces bacterial growth/survival in the lung by reducing glucose availability in the ASL. Dapagliflozin reduced blood and BALF glucose without negatively impacting lung glucose transport, fluid absorption or inflammatory responses in the lung. This is the first study to show that a reduction of blood glucose in hyperglycaemia has a direct beneficial effect on respiratory infection *in vivo.* These findings are relevant to the management and treatment of the diabetic exacerbation of respiratory disease, particularly in the light of increasing bacterial resistance to antibiotic therapy.

## Author contributions

D.B., E.B., J.T., J.G. and A.A. were responsible for study concept. A.A., C.W., A.B., J.T., H.G., S.G., M.O.W., D.W., A.L. and D.S. carried out the studies and analysed data. D.B. collated data and prepared manuscript with input from A.A., E.B., J.T. and J.G.

## Conflict of interest

The authors declare no conflicts of interest.

## Declaration of transparency and scientific rigour

This Declaration acknowledges that this paper adheres to the principles for transparent reporting and scientific rigour of preclinical research recommended by funding agencies, publishers and other organisations engaged with supporting research.
